# Supplementary material for: Targeting SARS-CoV-2 receptor-binding domain to cells expressing CD40 improves protection to infection in convalescent macaques
Source: Nat Commun. 2021 Sep 1;12:5215. doi: 10.1038/s41467-021-25382-0 (PMC8410935; doi:10.1038/s41467-021-25382-0)
Supplement: Supplementary file 1 — Supplementary information [file 41467_2021_25382_MOESM1_ESM.pdf]

Targeting SARS-CoV-2 receptor-binding domain to cells expressing CD40 improves protection to infection in convalescent macaques

Supplementary information

Supplementary figures

Supplementary Figure 1

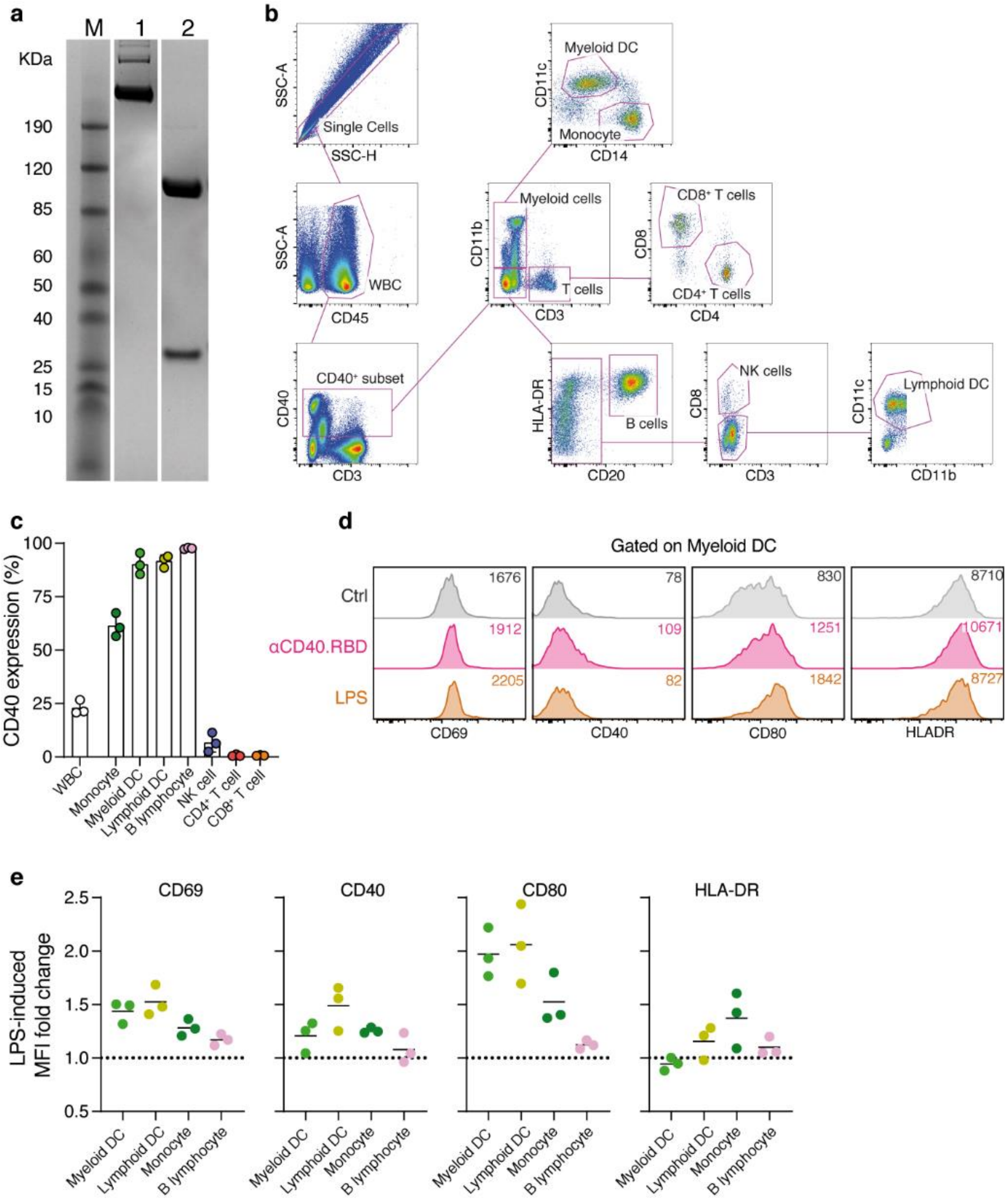

Supplementary Figure 1. CD40 expression on macaque PBMC. (**a**) SDS-PAGE analysis (representative of n=2 experiments). Lane M, BenchMark prestained protein ladder; lane 1, non-reduced and lane 2, reduced Coomassie blue stained SDS-PAGE analysis of anti-CD40 12E12-RBD. (**b**) Gating strategy to analyze the cell phenotype of the CD40⁺ fraction in PBMCs representative of a naive NHP (n = 3). (**c**) Percentage of expression of CD40 (12E12) on each cell subset. Bar indicates mean+/-SD of three animals. (**d**) Expression of activation markers after an 18-h incubation of NHP PMBC with culture medium (Ctrl, grey), αCD40.RBD (pink) or LPS (orange), considered as positive control, on the myeloid DC in representative naïve NHP (MF21). MFI values are indicated for each condition. (**e**) Fold change of MFI of activation markers after stimulation of NHP (n=3) PBMC with LPS on the myeloid DC, lymphoid DC, monocyte and B lymphocyte subsets. Bars indicate the mean values.

Supplementary Figure 2

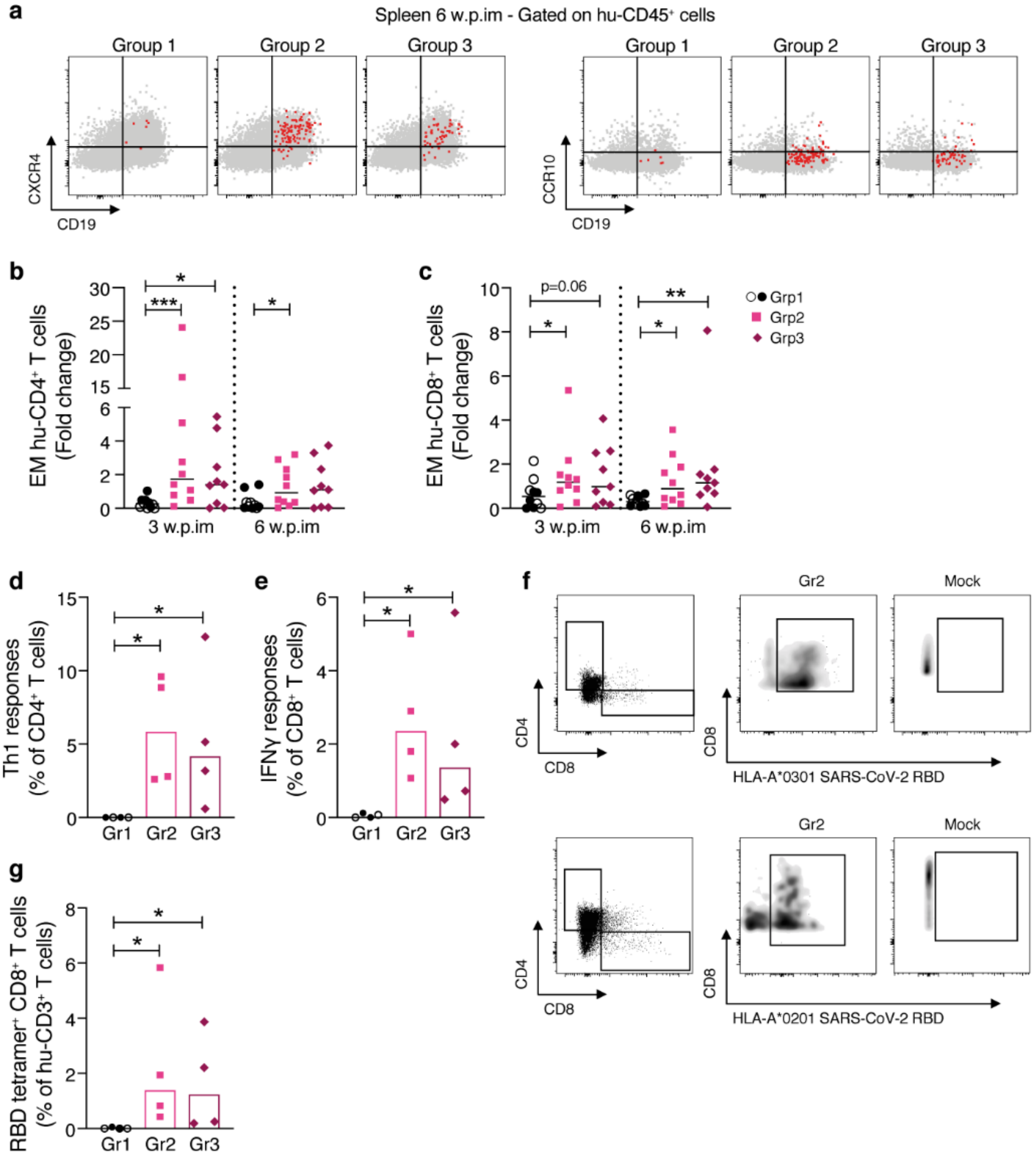

Supplementary Figure 2. Memory T- and B-cell responses elicited by the αCD40.RBD vaccine in hu-mice. (**a**) Manual back-gating of SARS-CoV-2 S protein-specific IgG-switched human B cells onto hCXCR4 versus hCD19 and hCCR10 versus hCD19 dot blots prepared from concatenated data from all hu-mice in each group. Bulk human B cells are colored in grey and S-specific IgG^+ human B cells in red. (**b**) Induction of effector memory $CD4^+$ and (**c**) $CD8^+$ T cells in hu-mouse blood by the vaccination regimens. Results are expressed as fold change at 3 and 6 w.p.im compared to baseline values. (**b-c**) Individual values are presented, along with the median (9 to 10 animals/group). (**d**) Frequency of antigen-specific Th1 human $CD4^+$ T cells (IFNγ+/- IL2+/- TNFα) and (e) antigen-specific human $CD8^+$ T cells ($IFN\gamma^+$) in the total human $CD4^+$ T-cell or human $CD8^+$ T-cell population, respectively, for non-immunized (mock) and vaccinated hu-mice. (**d-e**) Only pooled splenocytes from two or three HLA-A*0201 or HLA-A*0301 hu-mice were used due to low number of recovered cells. Each value is

presented, along with the median (4 determinations/group). The frequencies of IL-2^{+} antigen-specific hu-CD8^{+} T cells were 0% [0-0.72] (median, IQR; 25%-75%) for Gr2 and 0.02% [0-0.06] for Gr3, with no statistical difference between the two groups. The frequencies of TNF^{+} cells were 1.48% [0.79-5] for Gr2 and 0.83% [0.46-2.35] for Gr3, again without any statistical difference between the two groups. (**f**) Representative flow-cytometry plots showing the HLA-A*0301 and HLA-A*0201 tetramer^{+} SARS-CoV-2 RBD-protein-specific human CD8^{+} T cells for poly(IC)-injected hu-mice and αCD40.RBD-vaccinated hu-mice (group 2). (**g**) Frequency of RBD-specific human CD8^{+} T cells (Tetramer^{+}) in the total human T cell population for non-immunized (mock) and vaccinated hu-mice. Empty circle: PBS group; Full circle: poly:IC group; square: αCD40.RBD homologous prime/boost (group 2); Diamond: αCD40.RBD heterologous prime/boost (group 3). Only pooled splenocytes from two or three HLA-A*0201 or HLA-A*0301 hu-mice were used due to low number of recovered cells. Values are presented, along with the median (4 determinations/group). Mann-Whitney U-tests were used for comparisons. *$p < 0.05$, **$p<0.01$, ***$p<0.001$.

Supplementary Figure 3

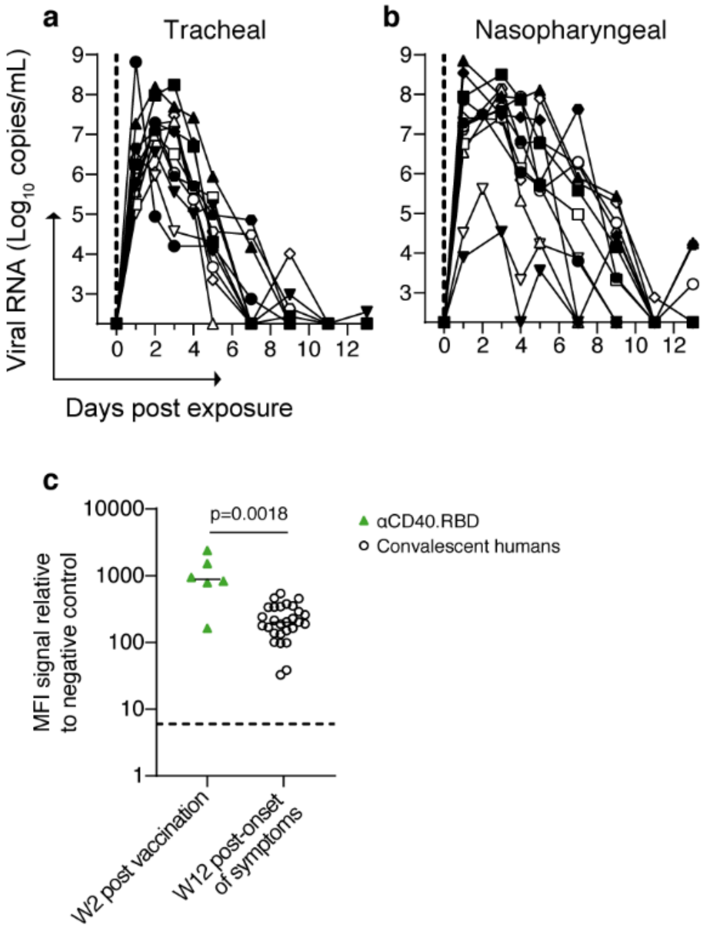

Supplementary Figure 3. Viral kinetics of first SARS-CoV-2 infection in NHPs. (**a-b**) Genomic viral RNA (gRNA) quantification in tracheal (**a**) and nasopharyngeal (**b**) swabs of macaques (n = 12) during the first SARS-CoV-2 infection 26 weeks prior to vaccination. Each line indicates individual values. (**c**) SARS-CoV-2 S protein-specific binding at week 2 post αCD40.RBD vaccination of macaques (n = 6) relative to that of convalescent humans (n = 28) sampled 12 weeks after the onset of symptoms. Horizontal dotted lines represent the background threshold. Bars indicate the median values for each group.

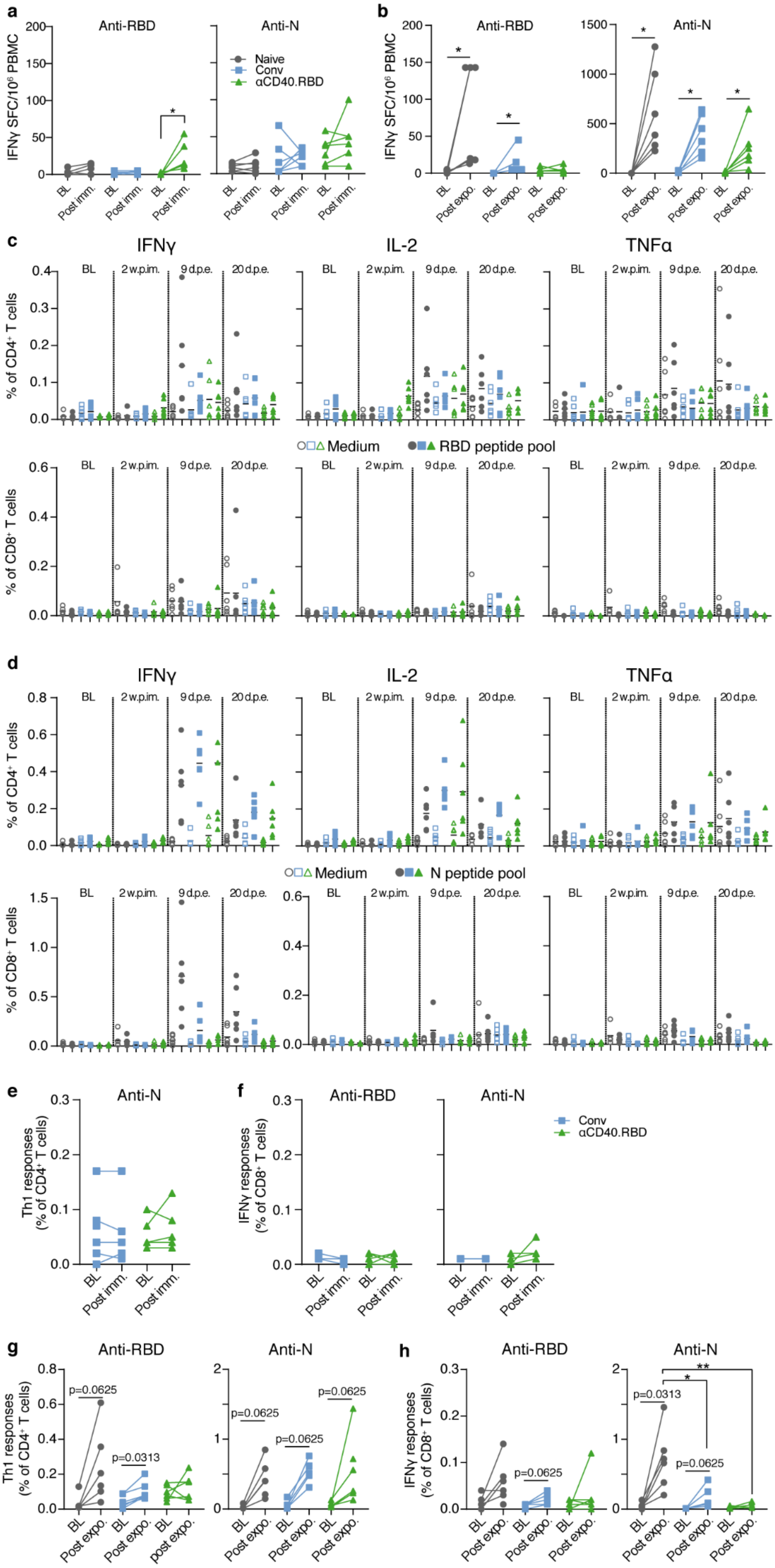
a
Anti-RBD
Anti-N
IFNγ SFC/10⁶ PBMC
Naive
Conv
αCD40.RBD
BL
Post imm.
*
b
Anti-RBD
Anti-N
Post expo.
c
IFNγ
IL-2
TNFα
2 w.p.im.
9 d.p.e.
20 d.p.e.
% of CD4⁺ T cells
% of CD8⁺ T cells
Medium
RBD peptide pool
d
N peptide pool
e
Th1 responses (% of CD4⁺ T cells)
f
IFNγ responses (% of CD8⁺ T cells)
g
p=0.0625
p=0.0313
post expo.
h
**

Supplementary Figure 4. Antigen-specific T-cell responses in NHPs. (**a-b**) Impact of vaccination (**a**) and SARS-CoV-2 exposure (**b**) on the number of IFNγ-secreting cells (n = 6). These cells were analyzed by ELISPOT after ex vivo stimulation with SARS-CoV-2 RBD or N overlapping peptide pools and plotted as spot-forming cells (SFC) per 1.0×10^6 PBMCs. the timepoints in each experimental group were compared using the Wilcoxon signed rank test (* two-tailed p=0.0313). (**a**) BL: Baseline approximately 1 week before immunization; “Post imm.”: Two weeks post immunization. (**b**) BL: Baseline the day of challenge; “Post expo.” Day 9 post SARS-CoV-2 challenge. (**c-d**) Frequency of IFNγ$^+$ (left), IL-2$^+$ (middle) or TFNα$^+$ (right) antigen-specific CD4$^+$ T cells (CD154$^+$) and CD8$^+$ T cells (CD137$^+$) in the total CD4$^+$ T cell (top) or CD8$^+$ T cell (bottom) population, respectively, for each naive macaque (grey symbols, n = 6), non-immunized convalescent macaque (blue symbols, n =6), and αCD40.RBD-vaccinated convalescent macaque (green symbols, n = 6). PBMCs were stimulated overnight with medium (open symbols), SARS-CoV-2 RBD (**c**) or N (**d**) overlapping peptide pools (filled symbols). BL: Baseline approximately 1 week before immunization. Bars indicate the mean values for each stimulation. (**e-h**) Frequency of antigen-specific Th1 CD4$^+$ T cells (CD154$^+$ and IFNγ +/- IL2 +/- TNFα) and antigen-specific CD8$^+$ T cells (CD137$^+$ IFNγ$^+$) in the total CD4$^+$ T cell (**e and g**) or CD8$^+$ T cell (**f and h**) population respectively. PBMC were stimulated overnight with SARS-CoV-2 RBD or N overlapping peptide pools. Time points in each experimental group were compared using the Wilcoxon signed rank test. (**e-f**) BL: Baseline approximately 1 week before immunization; “Post imm.”: Two weeks post immunization. (**g-h**) BL: Baseline approximately 2 weeks before challenge; “Post expo.” Day 9 post SARS-CoV-2 challenge. Groups were compared using the non-parametric Mann-Whitney test (*two-tailed p=0.0152, **two-tailed p=0.0022).

Supplementary Figure 5

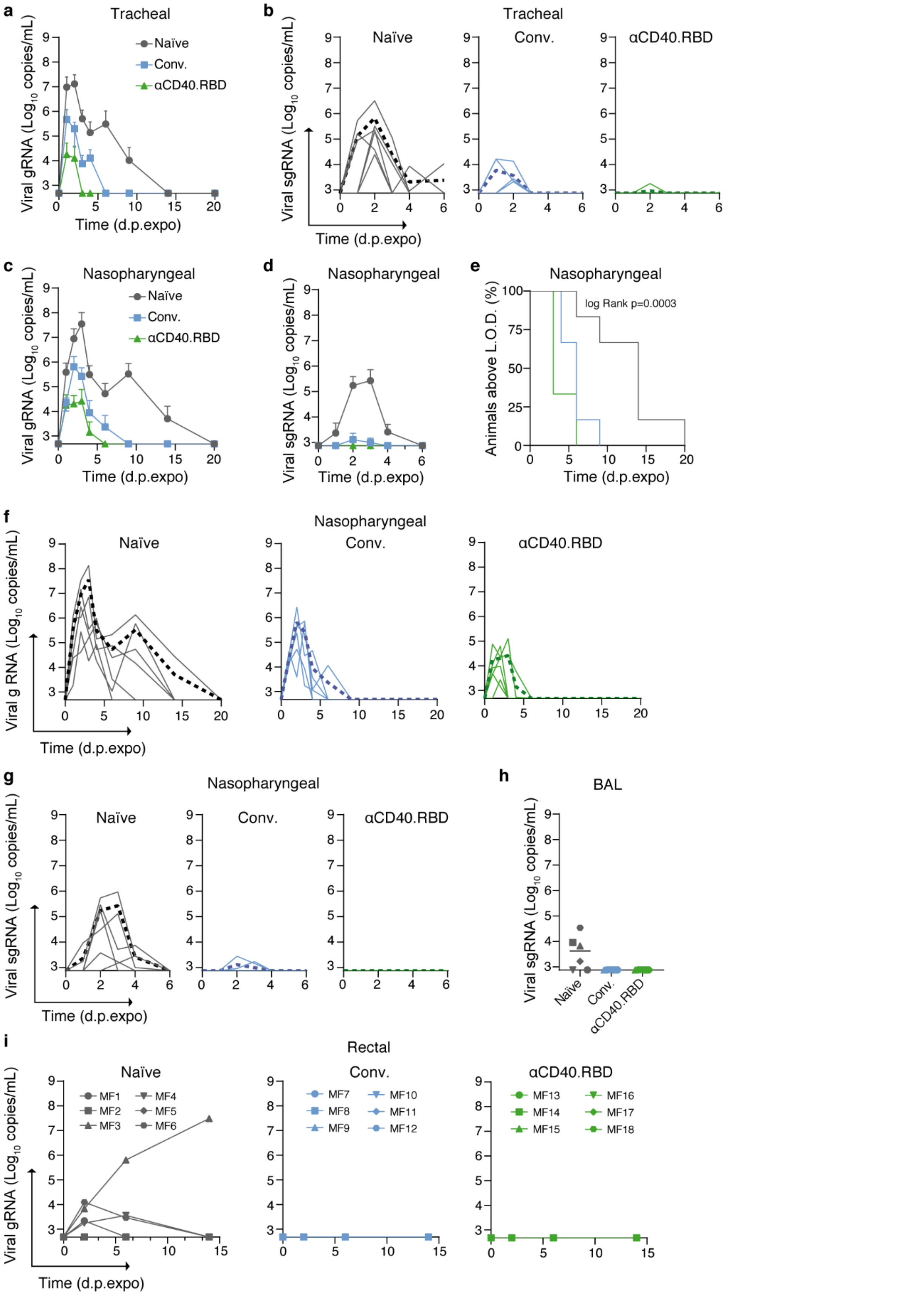

Supplementary Figure 5. SARS-CoV-2 viral kinetics in NHPs. gRNA and sgRNA viral loads in tracheal (**a-b**), nasopharyngeal (**b, c, d, f, g**), and rectal (**i**) swabs and BAL (**h**) in naive (grey, n = 6), convalescent

(blue, n = 6), and αCD40.RBD-vaccinated convalescent macaques (green, n = 6). (**b, f, g**) Each plain line indicates individual values, and the bold dotted lines represent the mean for each experimental group. (**a, c, d**) The means with the SD are shown. (**e**) Percentage of macaques with viral gRNA above the limit of detection over time in nasopharyngeal swabs. Experimental groups were compared using the log Rank test.

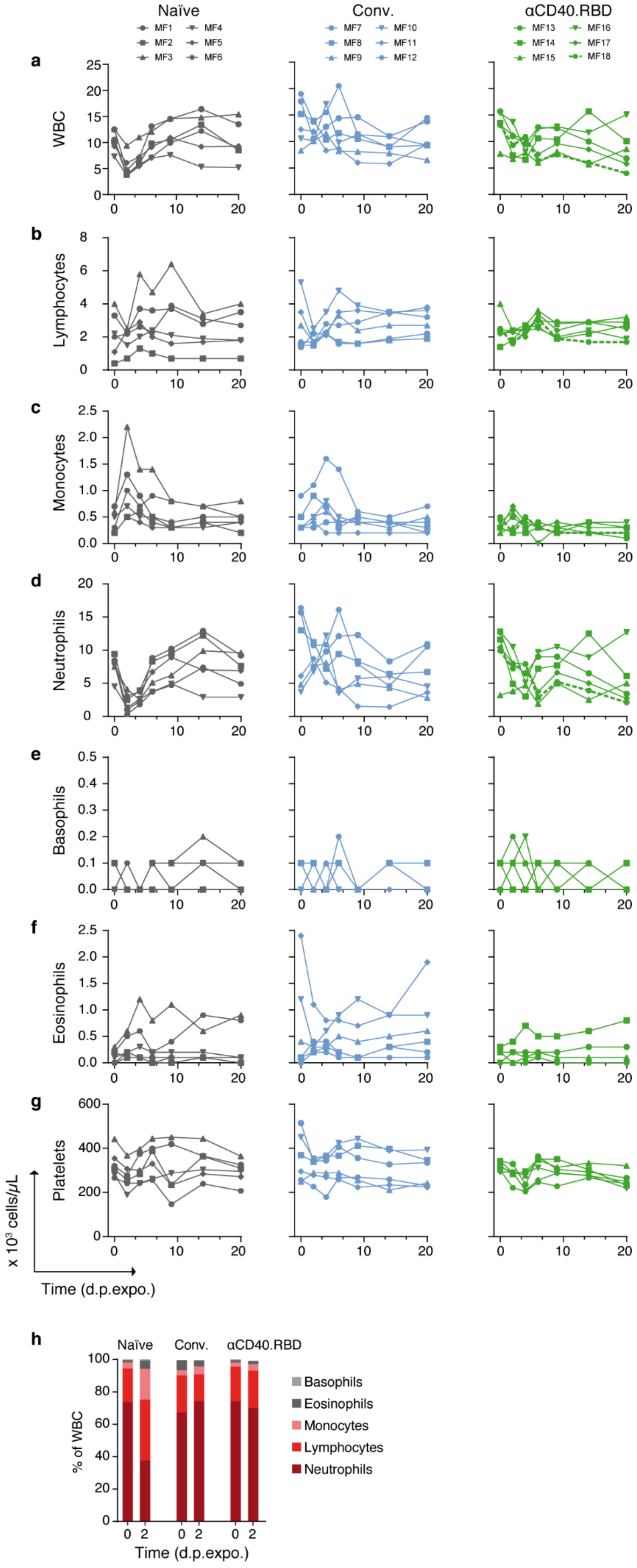
Naïve
Conv.
αCD40.RBD
MF1 MF2 MF3 MF4 MF5 MF6
MF7 MF8 MF9 MF10 MF11 MF12
MF13 MF14 MF15 MF16 MF17 MF18
a
WBC
b
Lymphocytes
c
Monocytes
d
Neutrophils
e
Basophils
f
Eosinophils
g
Platelets
x 10³ cells/μL
Time (d.p.expo.)
h
% of WBC
Basophils
Eosinophils
Monocytes
Lymphocytes
Neutrophils
Time (d.p.expo.)

Supplementary Figure 6. Complete blood counts of SARS-CoV-2 exposed naive and convalescent macaques. Absolute numbers of white blood cells (WBC) (**a**), lymphocytes (**b**), monocytes (**c**), neutrophils (**d**), basophils (**e**), eosinophils (**f**), and platelets (**g**) in naive (grey), convalescent (blue), and αCD40.RBD-vaccinated convalescent macaques (green) after SARS-CoV-2 exposure. (**h**) Frequency of cell subsets within WBC at days 0 and 2 post exposure according to NHP group. Each bar indicates the mean of 6 NHPs.

Supplementary Figure 7

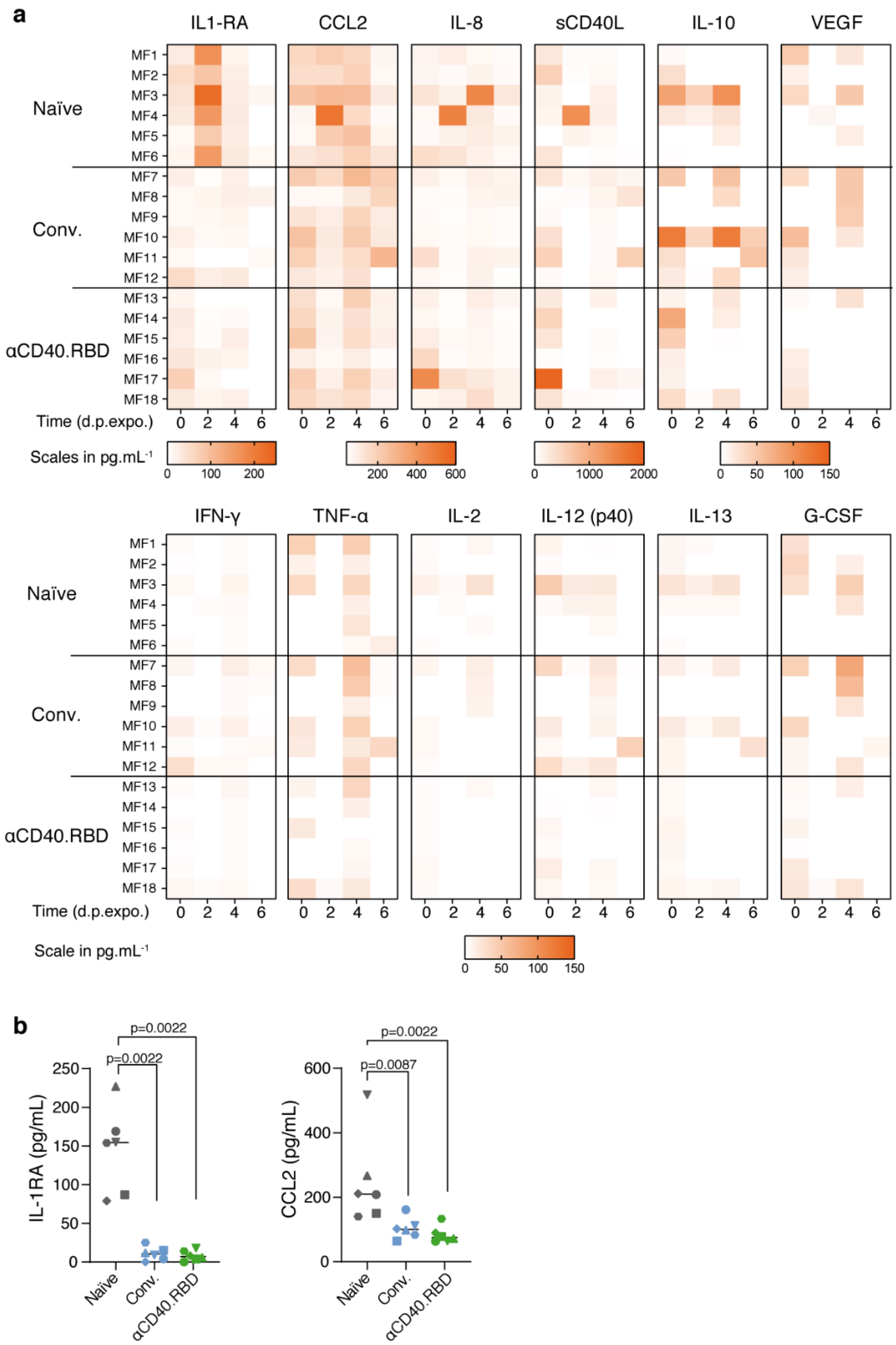

Supplementary Figure 7. Cytokines and chemokines in the plasma of naive and convalescent macaques after SARS-CoV-2 exposure. (**a**) Heatmaps of the plasma concentrations of 12 cytokines and chemokines. Each column represents one cytokine or chemokine. The color scale (in pg.mL^{-1}) is shown at the bottom. (**b**) Concentration of IL-1RA (left) and CCL2 (right) at day 2 post-exposure in plasma of naive (n=6, grey), convalescent (n=6, blue), and αCD40.RBD-vaccinated convalescent macaques (n=6, green). Each plot represents one macaque and bars represent median value for each group. Groups were compared using the non-parametric Mann-Whitney test (* Two-tailed $p < 0.05$).

Supplementary Figure 8

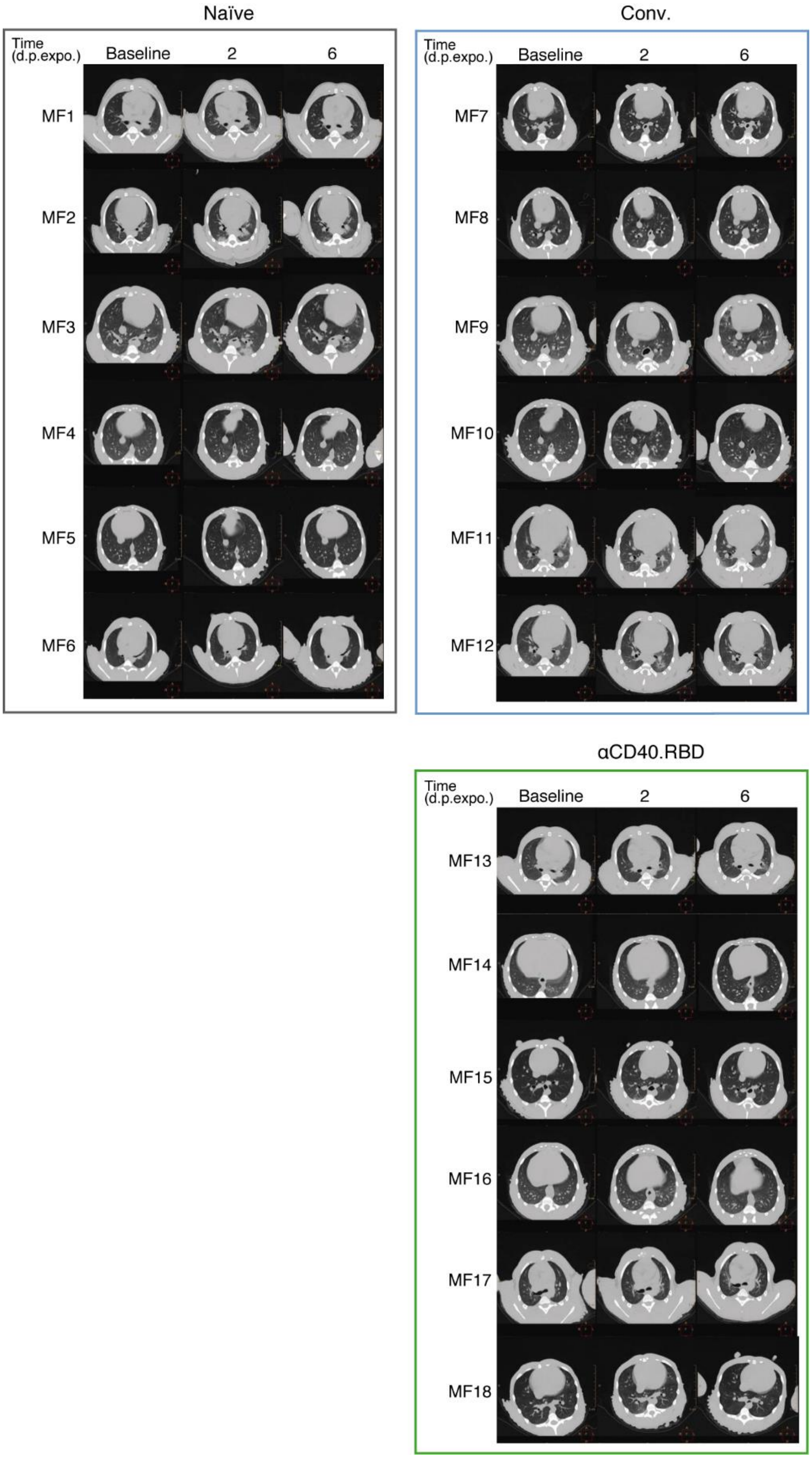

Supplementary Figure 8. Representative transversal slices of lung CT scans from SARS-CoV-2-exposed naive and convalescent macaques. Imaging was performed at baseline and days 2 and 6 post-exposure. Images are presented for each macaque according to their experimental group, with a window level of -300 and a window width of 1,600.

Supplementary Figure 9

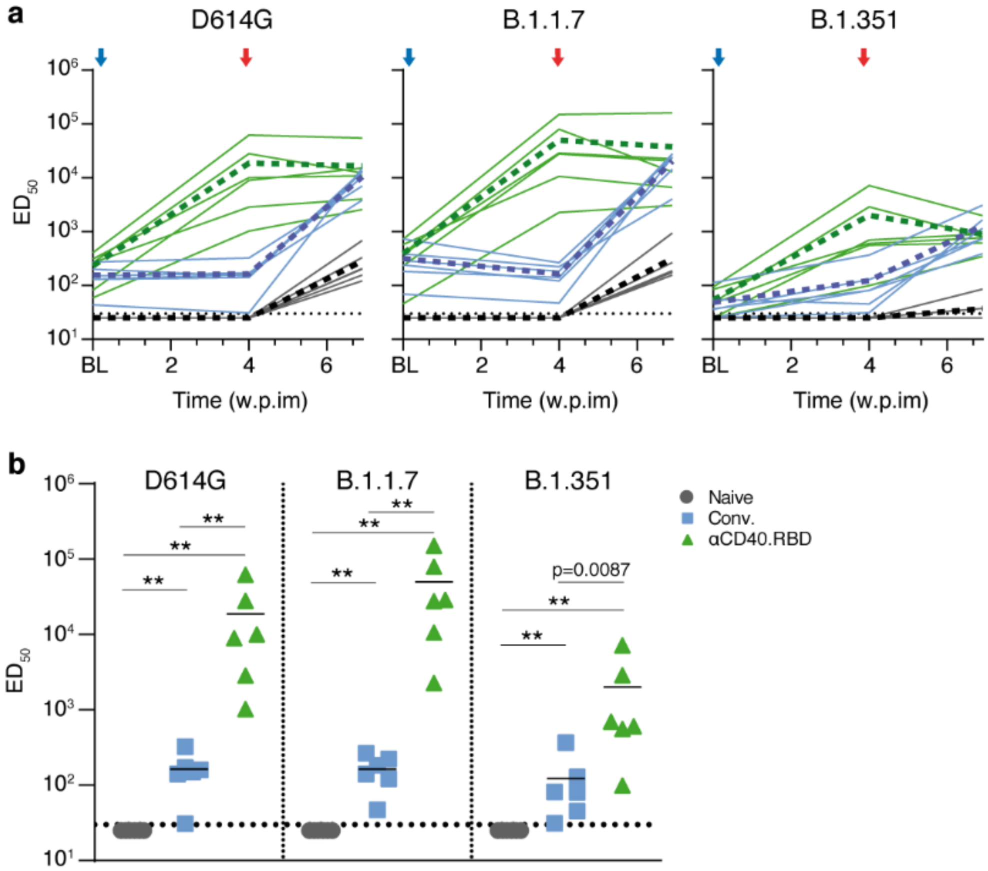

Supplementary Figure 9. Sensitivity of SARS-CoV-2 D614G, B.1.1.7 and B.1.351 variants to sera from NHPs. (**a**) Evolution overtime of neutralizing antibody titers in naive macaque (grey lines), non-immunized convalescent macaque (blue lines), and αCD40.RBD-vaccinated convalescent macaque (green lines). Each plain line indicates the individual values, and the bold dotted lines represent the mean for each experimental group. Horizontal dotted line indicates the limit of detection (ED50 = 30). The blue and red arrows indicate vaccination and viral exposure, respectively. (**b**) Neutralization ED50 values of the three viral isolates at the day of challenge (4 w.p.im). Each plot represents one macaque (n=6 NHP/group) and bars indicate mean value for each group. Groups were compared using the non-parametric Mann-Whitney test (** Two-tailed p=0.0022).

Supplementary Figure 10

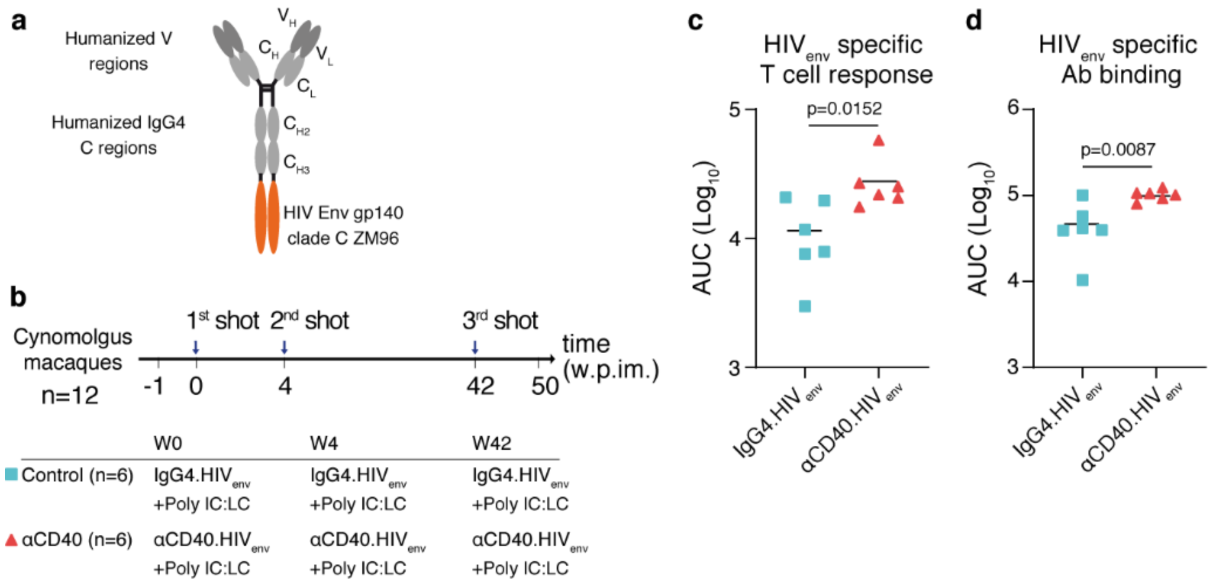

Supplementary Figure 10. Comparison of immunogenicity of αCD40.HIVenv vaccine to the non-targeting isotype control IgG4.HIVenv in cynomolgus macaques. (**a**) Picture of αCD40.HIVenv vaccine construct. (**b**) Study design including three injections of the vaccines (200µg by subcutaneous route) co-injected with poly-IC-LC (Iltonol, 1 mg by subcutaneous route). (**c**) HIV-Env specific response as measures by IFNγ ELISPOT following overnight stimulation of NHP PBMC with a pool of 15-mer overlapping peptides spanning the Env gp140. Area under the curve (AUC) from day 0 to day 50 of the immunization. (**d**) Anti- HIV-Env IgG in serum of immunized macaques. Area under the curve from day 0 to day 50 of the immunization. (**c-d**) Each plot represents one macaque (n=6 NHP/group). The mean of 6 NHPs is indicated by horizontal line. Groups were compared using the non-parametric Mann-Whitney test (Two-tailed p values are indicated).

Supplementary Figure 11

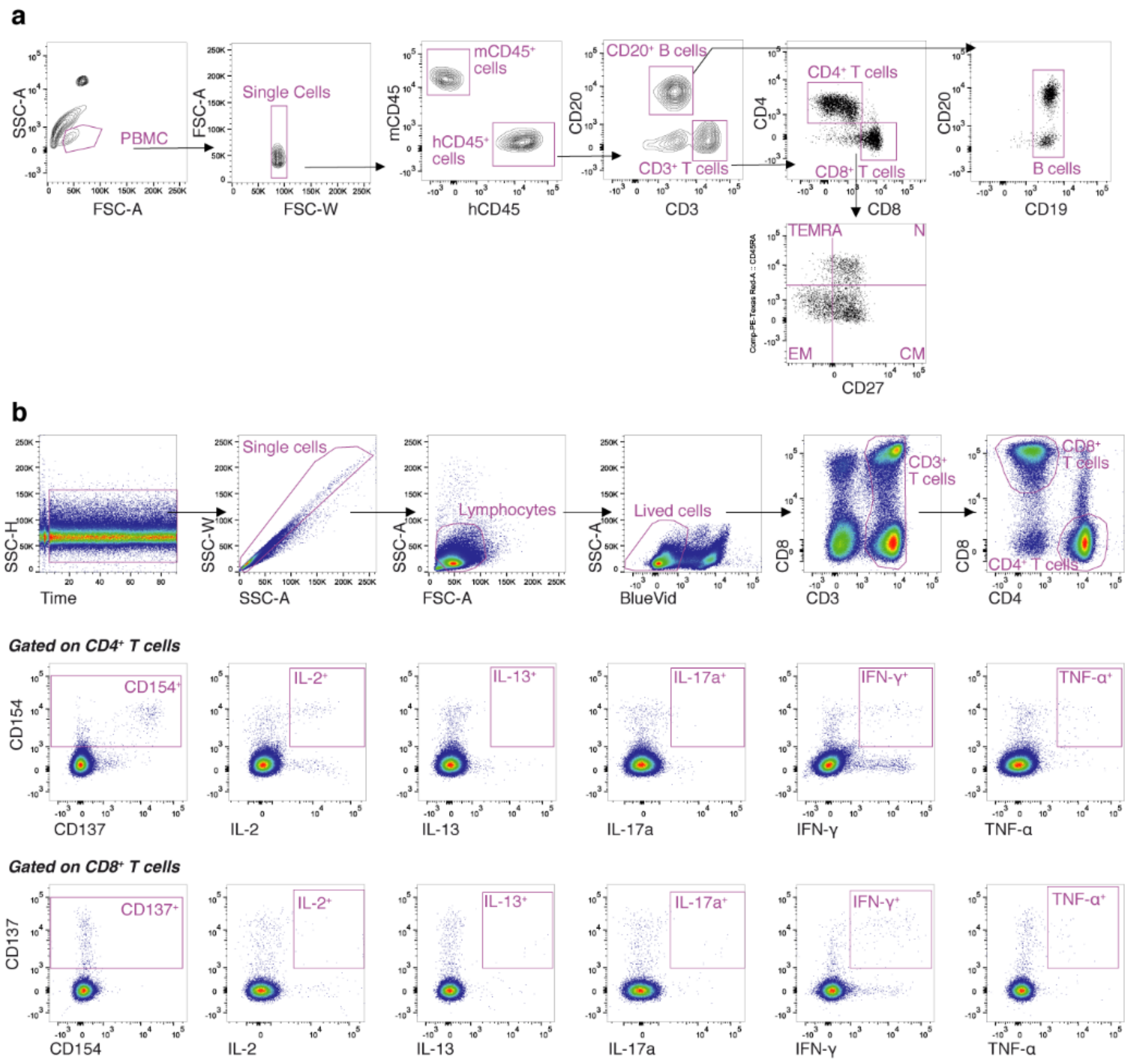

Supplementary Figure 11. Gating strategy for flow cytometry analysis. (**a**) Gating strategy to analyze B cells and T cells in hu-mice experiments presented on Fig. 1f-k and on supplementary Fig. 2. (**b**) Gating strategy to analyze antigen-specific T-cell responses in NHPs presented on Fig. 2f-g and on supplementary Fig.4c-h.

Supplementary Tables

Supplementary Table I. HLA class 1 phenotype of donors who provided the stem cells for hu-mouse reconstitution.

Donnor	HLA-A class I	Grp 1	Grp 2	Grp 3
2587	A*01:01	2	2	1
2626	A*02:02	4	3	4
5855	A*25:01	0	1	0
5898	ND	1	0	0
8035	A*03:01	3	4	4

Supplementary Table II. Names and sequences of primers.

Name	Sequences (5'-3')
SARS-CoV-2 E gene subgenomic mRNA	
leader-specific primer sgLeadSARSCoV2-F	CGATCTCTTGTAGATCTGTTCTC
E-Sarbeco-R primer	ATATTGCAGCAGTACGCACACA
E-Sarbeco probe	ACACTAGCCATCCTTACTGCGCTTCG [5']HEX [3']BHQ-1
RdRp gene / nCoV_IP4	
nCoV_IP4-14059Fw	GGTAACTGGTATGATTTCG
nCoV_IP4-14146Rv	CTGGTCAAGGTTAATATAGG
nCoV_IP4-14084Probe(+)	TCATACAAACCACGCCAGG [5']Fam [3']BHQ-1

Supplementary Methods

αCD40.RBD vaccine

Production and quality assurance of the αCD40.RBD vaccine Vectors and sequences for humanized anti-human CD40 12E12 IgG4 and control human IgG4 antibodies have been described previously [1-3]. GenBank sequences HQ738666.1 and KP684037 describe the human IgG4 chimeric forms of the 12E12, anti-human CD40 H and L chains. Methods for expression vectors and protein production and purification, via transient or stable CHO-S (Chinese Hamster Ovary cells; ThermoFisher Scientific) transfection and quality assurance including CD40 binding specificity were as are described. CHO-optimized codons encoding SARS-CoV-2 RBD residues 318-541 of sequence ID: YP_009724390.1 with appended residues encoding a C-tag (EPEA) and a stop codon were inserted between the vector *Nhe* I and *Not* I sites positioned distal to the H chain C-terminal codon. Expression plasmids encoding the antibody H chain RBD fusion and the L chain were transiently transfected into Expi-CHO cells (ThermoFisher Scientific) with TransIT-PRO Pro reagent (Mirus Bio) using the manufacturers protocol. The product was purified by protein A affinity capture of the culture medium followed by elution with a gradient of 1M L-Arginine monohydrochloride in H2O, from pH 8.0 and pH 1.8. Product was formulated in phosphate buffered saline (pH 7.4) with 125 mM cyclodextrin (average MW 1420). The LPS value was .037 ng/mg. Using a solid phase assay direct binding assay previously described [3] these was no significant difference in the CD40 binding affinity of anti-CD40 12E12 (EC50 30 pM) versus anti-CD40 12E12-RBD (EC50 35 pM).

DREP-S vaccine

DREP-S vaccine constructs were made by cloning the sequences encoding S of SARS-CoV-2 spike protein into the Semliki Forest Virus (SFV) DREP plasmid vector backbone 3 using BamHI and SpeI restriction sites [4]. The S construct encodes the surface glycoprotein of SARS-CoV-2 (Wuhan-Hu-1) with an 18-aa deletion in the cytoplasmic tail (D18). The synthesis of the construct with the appropriate restriction sites was ordered from Twist bioscience. The spike variant was codon optimized for human expression and the construct's sequence was confirmed by sequencing. Plasmid DNA of the DREP-S vaccine candidate was purified from bacterial cultures using the EndoFree Plasmid Maxi or Giga Kit (QIAGEN) and the concentration and purity was measured on a NanoDrop One (ThermoFisher).

Binding of αCD40.RBD vaccine to non-human primate cells and activation PBMC assays

PBMC from 3 naïve macaques were isolated and stained for 15 min with anti-CD11b-V450 (1:30; # 560480; ICRF44, BD), anti-CD3-V500 (1:20; # 560770; SP34-2, BD), anti-CD11c-BV605 (1:20; # 301636; 3.9, BioLegend), anti-CD8-BV650 (1:50; #563821; BW135/80, Miltenyi Biotec), anti-CD20-BV711 (1:20; # 563126; 2H7, Invitrogen), anti-CD163-APC (1:20; #333610; GHI/61, BioLegend), anti-CD14-A700 (1:25; # 301822; M5E2, BioLegend), anti-HLA-DR-APC-H7 (1:40; # 641411; L243, BD), anti-CD4-FITC (1:5; # 550628; L200, BD), anti-CD45-PerCP (1:40; # 558411; D058-1283, BD) and anti-CD40-AF594 (12E12) or αCD40.RBD-AF594. Next, cells were washed twice with PBS and acquired on the ZE5 flow cytometer (Biorad). Moreover, a part of these PMBCs were also incubated 18 hours with culture medium (RPMI 1640 media with L-Glutamax supplemented with Penicillin / Streptomycin and 10% of fetal calf serum (FBS)) and stimulated with αCD40.RBD (10 µg/mL) or LPS (100 ng/mL, Invivogen). Next, cells were washed in PBS and incubated 15min with LIVE/DEAD fixable Blue Dead Cell marker (Life Technologies), anti-CD11b-V450 (1:30; # 560480; ICRF44, BD), anti-CD3-V500 (1:20; # 560770; SP34-2, BD), anti-CD86-BV605 (1:20; #562999; 2331, BD), anti-CD11c-APC (1:20; #301614; 3.9, BioLegend), anti-CD20-BV711 (1:20; # 563126; 2H7, Invitrogen), anti-CD80-BV786 (1:20; #563821; L307.4, BD), anti-CD8-BV650 (1:50; #563821; BW135/80, Miltenyi Biotec), anti-CD14-A700 (1:25; #301822; M5E2, BioLegend), anti-HLA-DR-APC-H7 (1:40; # 641411; L243, BD), anti-CD4-FITC (1:5; # 550628; L200, BD),

anti-CD45-PerCP (1:40; # 558411; D058-1283, BD), anti-CD69-PE-Cy7 (1:10; #557745; FN50, BD) and anti-CD40-AF594 (12E12). Next, cells were washed twice with PBS and acquired on the ZE5 flow cytometer (Biorad). Analysis was performed on FlowJo v.10 software. For activation markers, results were expressed as fold change geometric MFI, obtained by dividing the geometric MFI measure in αCD40.RBD or LPS stimulation by the background geometric MFI measure in control stimulation (incubation with medium only).

T-cells response in hu-mice

To analyze the SARS-CoV-2 RBD protein-specific T cell using functional recall assay, we used fifteen-mer peptides (n = 70) overlapping by 11 amino acids (aa) and covering the vaccine RBD sequence (aa281-571 from Spike) synthesized by JPT Peptide Technologies (Berlin, Germany) and used at a final concentration of 1 µg/mL. We also used HLA class I PE labelled tetramers purchased from ProImmune Ltd (Oxford, UK). We used the following two specificities: SARS-CoV-2 A*0201 KIA (KIADYNYKL), SARS-CoV-2 A*0301 KCY (KCYGVSPTK).

Cryopreserved hu-mice spleen cells from 6 weeks after the priming immunization (one week after final immunization) were thawed and counted. Cells were rested overnight in RPMI 1640 media with L-Glutamax supplemented with Penicillin / Streptomycin and 10% of human serum. Subsequently, cells from HLA-A*0201 and HLA-A*0301 donors were pooled together for the mock group and group 2 plus 3 vaccinated hu-mice, then cultured at $0.6x10^6$ cells per condition with 1µg/mL of 15-mers peptides JPT Peptide Technologies (Berlin, Germany). As a negative control no stimulant was added, and as a positive control 1 µL of DynabeadsTM CD3/CD28 (ThermoFischer Scientific) were used. IL-2 (100 IU/mL, R&D System) was added on day 2, half of the volume of each culture well was refreshed with fresh media containing IL-2 (10 U/mL) at day 5 and with fresh media without IL-2 at day 7. On day 8, cells were re-stimulated: no stimulant was added in the negative control, 100 ng/mL Staphylococcal enterotoxin B (LL-122, Cliniscience) was added in the positive control and 15-mers peptides in the condition of interest. BD GolgiPlug (Becton Dickinson France) was added in all conditions and the culture was continued for additional 18 hours. Next, spleen cells were washed using FACS buffer (PBS, supplemented with 1% FBS) and incubated with tetramer-PE (ProImmune Ltd, Oxford, UK), Live dead fixable Aqua Dead marker (Life Technologies) and the following antibodies: anti-hCD3-A700 (1:50, # 2102120, UCHT1, Sony), anti-h-CD4-BV605 (1:10, # 2102780, RPA-T4, Sony), anti-hCD8-APC-Cy7 (1:10, # 2323570, SK1, Sony) for 30 minutes. Following fixation and permeabilization, spleen cells were stained with intracellular antibodies: anti-hIFNγ-PerCPCy5.5 (1:10, # 3132640, B27, Sony), anti-hIL-2-BV421 (1:16, # 3101640, MQ1-17H12, Sony), anti-hTNFα-PC7 (1:10, # 3114650, Mab11, Sony) for 30 minutes. Stained cells were acquired on the LSRII flow cytometer (BD Biosciences). FlowJo v.10.7.1 software was used for data analysis (TreeStar, Inc., Ashland, OR).

SARS-CoV-2 S protein-specific B cell analysis

Hu-mice PBMC from 3 weeks after the priming immunization and hu-mice PBMC and spleen cells from 6 weeks (one week after the last recall injection) were incubated first with the biotinylated SARS-CoV-2 S protein for 30 min at 4°C. After a washing step, cells were stained for 30 min at 4°C with streptavine-AF700 (1:10, ThermoFisher Scientific), anti-human (h) CD45-PeCy7 (1:50, # 2120080, HI30, Sony), anti-mouse (m) CD45-BV711 (1:50, # 1115735, 30F11, Sony), anti-hCXCR4-Pe-Dazzle (1:50, # 12-9999-42, 12G5, eBiosciences), anti-hCCR10-PE (1:50, #314305; R&D System), anti-CD3-BV510 (1:50, #2102240, UCHT1, Sony), anti-CD4-FITC (1:50, #2187040, OKT4, Sony), anti-CD8-PerCpCy5.5 (1:50, #2323550, SK1, Biolegend) antibodies and the following B cell-specific antibodies: anti-hCD19-BV421 (1:16, #2111170, HIB19, Sony), anti-hCD20-APC (1:50, #2111550, 2H7, Sony), anti-hIgG-BV786 (1:16, #564230, G18-145, BD Biosciences), anti-hCD38-APC-Cy7 (1:16, # 2117670, HIT2, Sony).. Staining on

spleen cells also included a viability marker (LiveDead aqua or yellow stain ThermoFisher Scientific). Cells were washed twice with FACS buffer (PBS 1% FCS) and acquired on the LSRII flow cytometer (BD Biosciences). Analyses were performed on FlowJo v.10.7.1.

Virus neutralization assays

Virus strains: The reference D614G strain (hCoV-19/France/GE1973/2020) was supplied by the National Reference Centre for Respiratory Viruses hosted by Institut Pasteur and headed by S.v.d.W. This viral strain was supplied through the European Virus Archive goes Global (EVAg) platform, a project that has received funding from the European Union's Horizon 2020 research and innovation program under grant agreement number 653316. The variant strains were isolated from nasal swabs on Vero cells and amplified by one or two passages on Vero cells. The B.1.1.7 strain originated from an individual in Tours (France) who returned from the United Kingdom. The B.1.351 strain (CNR 202100078) originated from an individual in Créteil (France). Both individuals provided informed consent for the use of their biological materials. Titration of viral stocks was performed on Vero E6 cells, with a limiting dilution technique allowing a calculation of the 50% tissue culture infectious dose, or on S-Fuse cells. Viruses were sequenced directly on nasal swabs and after one or two passages on Vero cells.

S-Fuse neutralization assay: The assay was performed as described [8]. U2OS-ACE2 GFP1–10 or GFP 11 cells (obtained from U2OS cells, ATCC HTB-96), also termed S-Fuse cells, become GFP^+ cells when they are productively infected with SARS-CoV-2 [9]. Cells were tested negative for mycoplasma. Cells were mixed (at a 1:1 ratio) and plated at 8×10^3 cells per well in a µClear 96-well plate (Greiner Bio-One). The indicated SARS-CoV-2 strains were incubated with sera at the indicated concentrations or dilutions for 15 min at room temperature and added to S-Fuse cells. Sera were heat inactivated 30 min at 56 °C before use. Then, 18 h later, cells were fixed with 2% paraformaldehyde, washed and stained with Hoechst (1:1,000 dilution; Invitrogen). Images were acquired with an Opera Phenix high-content confocal microscope (PerkinElmer). The GFP area, the number of syncytia and nuclei were quantified using the Harmony software (PerkinElmer). The percentage of neutralization was calculated using the number of syncytia as the value with the following formula: 100 × (1 − (value with serum − value in 'noninfected')/(value in 'no serum' − value in 'noninfected')). Neutralizing activity of each sera was expressed as the ED50.

Antigen specific T cell assays using non-human primate cells

To analyze the SARS-CoV-2 protein-specific T cell using functional assay, 15-mer peptides (n = 70) overlapping by 11 amino acids (aa) and covering the vaccine RBD sequence (n=70, aa 281-571 from Spike) and the SARS-CoV-2 Nucleoprotein sequence (n=102, aa 1-419 from N) synthesized by JPT Peptide Technologies (Berlin, Germany) and used at a final concentration of 2 µg/mL.

IFNγ ELISpot assay of PBMC was performed using the Monkey IFNγ ELISpot PRO kit (Mabtech Monkey IFNγ ELISPOT pro, #3421M-2APT) according to the manufacturer's instructions. PBMC were stimulated with RBD or N sequence overlapping peptide pools at a final concentration of 2 µg/mL. Plates were incubated for 18 h at 37°C in an atmosphere containing 5% CO_2, then washed 5 times with PBS and incubated for 2 h at 37°C with a biotinylated anti-IFNγ antibody. After 5 washes, spots were developed by adding 0.45 µm-filtered ready-to-use BCIP/NBT-plus substrate solution and counted with an

automated ELISpot reader ELRIFL04 (Autoimmun Diagnostika GmbH, Strassberg, Germany). Spot forming units (SFU) per 1.0×10^6 PBMC are means of duplicates for each animal.

T-cell responses were also characterized by measurement of the frequency of PBMC expressing IL-2 (PerCP5.5, 1:10; # 560708; MQ1-17H12, BD), IL-17a (Alexa700, 1:20; # 560613; N49-653, BD), IFN-γ (V450, 1:33.3; # 560371; B27, BD), TNF-α (BV605, 1:30.3; # 502936; Mab11, BioLegend), IL-13 (BV711, 1:20; # 564288; JES10-5A2, BD), CD137 (APC, 1:20; # 550890; 4B4, BD) and CD154 (FITC, 1:20; # 555699; TRAP1, BD) upon stimulation with the two peptide pools. CD3 (APC-Cy7, 1:200; #557757; SP34-2, BD), CD4 (BV510, 1:33.3; # 563094; L200, BD) and CD8 (PE-Vio770, 1:50; # 130-113-159; BW135/80, Miltenyi Biotec) antibodies was used as lineage markers. One million of PBMC were cultured in complete medium (RPMI1640 Glutamax+, Gibco; supplemented with 10 % FBS), supplemented with co-stimulatory antibodies (FastImmune CD28/CD49d, Becton Dickinson). Then cells were stimulated with S or N sequence overlapping peptide pools at a final concentration of 2 µg/mL. Brefeldin A was added to each well at a final concentration of 10µg/mL and the plate was incubated at 37°C, 5% CO2 during 18 h. Next, cells were washed, stained with a viability dye (LIVE/DEAD fixable Blue dead cell stain kit, ThermoFisher), and then fixed and permeabilized with the BD Cytofix/Cytoperm reagent. Permeabilized cell samples will be stored at -80 °C before the staining procedure. Antibody staining was performed in a single step following permeabilization. After 30 min of incubation at 4°C, in the dark, cells were washed in BD Perm/Wash buffer then acquired on the ZE5 flow cytometer (Biorad). Analysis was performed on FlowJo v.10 software.

Virus quantification in cynomolgus macaque samples

Upper respiratory (nasopharyngeal and tracheal) and rectal specimens were collected with swabs (Viral Transport Medium, CDC, DSR-052-01). Tracheal swabs were performed by insertion of the swab above the tip of the epiglottis into the upper trachea at approximately 1.5 cm of the epiglottis. All specimens were stored between 2°C and 8°C until analysis by RT-qPCR with a plasmid standard concentration range containing an RdRp gene fragment including the RdRp-IP4 RT-PCR target sequence (Supplementary table II). The limit of detection was estimated to be 2.67 $\log_{10}$ copies of SARS-CoV-2 gRNA per mL and the limit of quantification was estimated to be 3.67 $\log_{10}$ copies per mL. SARS-CoV-2 E gene subgenomic mRNA (sgRNA) levels were assessed by RT-qPCR using primers and probes previously described (Corman et al., 2020; Wölfel et al., 2020) (Supplementary table SII). The protocol describing the procedure for the detection of SARS-CoV-2 is available on the WHO website (https://www.who.int/docs/default-source/coronaviruse/real-time-rt-pcr-assays-for-the-detection-of-sars-cov-2-institut-pasteur-paris.pdf?sfvrsn=3662fcb6_2). The limit of detection was estimated to be 2.87 $\log_{10}$ copies of SARS-CoV-2 sgRNA per mL and the limit of quantification was estimated to be 3.87 $\log_{10}$ copies per mL[10,11].

Methods references

1 Flamar, A. L. *et al.* Targeting concatenated HIV antigens to human CD40 expands a broad repertoire of multifunctional CD4+ and CD8+ T cells. *Aids* **27**, 2041-2051, doi:10.1097/QAD.0b013e3283624305 (2013).

2 Li, D. *et al.* Targeting self- and foreign antigens to dendritic cells via DC-ASGPR generates IL-10-producing suppressive CD4+ T cells. *J Exp Med* **209**, 109-121, doi:10.1084/jem.20110399 (2012).

3 Zurawski, G. *et al.* Superiority in Rhesus Macaques of Targeting HIV-1 Env gp140 to CD40 versus LOX-1 in Combination with Replication-Competent NYVAC-KC for Induction of Env-Specific Antibody and T Cell Responses. *Journal of virology* **91**, doi:10.1128/jvi.01596-16 (2017).

4 Szurgot, I. *et al.* DNA-launched RNA replicon vaccines induce potent anti-SARS-CoV-2 immune responses in mice. *Scientific Reports* (2021).

5 Maisonnasse, P. *et al.* Hydroxychloroquine use against SARS-CoV-2 infection in non-human primates. *Nature*, doi:10.1038/s41586-020-2558-4 (2020).

6 Fenwick, C. *et al.* Changes in SARS-CoV-2 Spike versus Nucleoprotein Antibody Responses Impact the Estimates of Infections in Population-Based Seroprevalence Studies. *Journal of virology* **95**, doi:10.1128/jvi.01828-20 (2021).

7 Johnson, M. *et al.* Evaluation of a novel multiplexed assay for determining IgG levels and functional activity to SARS-CoV-2. *J Clin Virol* **130**, 104572, doi:10.1016/j.jcv.2020.104572 (2020).

8 Planas, D. *et al.* Sensitivity of infectious SARS-CoV-2 B.1.1.7 and B.1.351 variants to neutralizing antibodies. *Nature medicine*, doi:10.1038/s41591-021-01318-5 (2021).

9 Buchrieser, J. *et al.* Syncytia formation by SARS-CoV-2-infected cells. *Embo j* **40**, e107405, doi:10.15252/embj.2020107405 (2021).

10 Baccam, P., Beauchemin, C., Macken, C. A., Hayden, F. G. & Perelson, A. S. Kinetics of influenza A virus infection in humans. *Journal of virology* **80**, 7590-7599, doi:10.1128/jvi.01623-05 (2006).

11 Gonçalves, A. *et al.* Timing of Antiviral Treatment Initiation is Critical to Reduce SARS-CoV-2 Viral Load. *CPT Pharmacometrics Syst Pharmacol* **9**, 509-514, doi:10.1002/psp4.12543 (2020).
