# Supplementary material for: Targeting SARS-CoV-2 receptor-binding domain to cells expressing CD40 improves protection to infection in convalescent macaques
Source: Nat Commun. 2021 Sep 1;12:5215. doi: 10.1038/s41467-021-25382-0 (PMC8410935; doi:10.1038/s41467-021-25382-0)
Supplement: Supplementary file 3 — Reporting Summary [file 41467_2021_25382_MOESM3_ESM.pdf]

nature research

Corresponding author(s): Roger Le Grand

Last updated by author(s): Jul 8, 2021

Reporting Summary

Nature Research wishes to improve the reproducibility of the work that we publish. This form provides structure for consistency and transparency in reporting. For further information on Nature Research policies, see our Editorial Policies and the Editorial Policy Checklist.

Statistics

For all statistical analyses, confirm that the following items are present in the figure legend, table legend, main text, or Methods section.

n/a	Confirmed	
☐	☒	The exact sample size (*n*) for each experimental group/condition, given as a discrete number and unit of measurement
☐	☒	A statement on whether measurements were taken from distinct samples or whether the same sample was measured repeatedly
☐	☒	The statistical test(s) used AND whether they are one- or two-sided<br>*Only common tests should be described solely by name; describe more complex techniques in the Methods section.*
☐	☒	A description of all covariates tested
☐	☒	A description of any assumptions or corrections, such as tests of normality and adjustment for multiple comparisons
☐	☒	A full description of the statistical parameters including central tendency (e.g. means) or other basic estimates (e.g. regression coefficient) AND variation (e.g. standard deviation) or associated estimates of uncertainty (e.g. confidence intervals)
☐	☒	For null hypothesis testing, the test statistic (e.g. *F*, *t*, *r*) with confidence intervals, effect sizes, degrees of freedom and *P* value noted<br>*Give P values as exact values whenever suitable.*
☒	☐	For Bayesian analysis, information on the choice of priors and Markov chain Monte Carlo settings
☒	☐	For hierarchical and complex designs, identification of the appropriate level for tests and full reporting of outcomes
☒	☐	Estimates of effect sizes (e.g. Cohen's *d*, Pearson's *r*), indicating how they were calculated

Our web collection on statistics for biologists contains articles on many of the points above.

Software and code

Policy information about availability of computer code

Data collection	Data was collected using classical Excel Files. Macaque data were stored in a Laboratory Information Management System called BatLab.
Data analysis	Data was analyzed using GraphPad Prism v8, Flowjo V.10, Flowjo V.10.7.1, Microsoft Excel 2016 (16.0.5173.1000), Harmony v4.9.

For manuscripts utilizing custom algorithms or software that are central to the research but not yet described in published literature, software must be made available to editors and reviewers. We strongly encourage code deposition in a community repository (e.g. GitHub). See the Nature Research guidelines for submitting code & software for further information.

Data

Policy information about availability of data

All manuscripts must include a data availability statement. This statement should provide the following information, where applicable:

- Accession codes, unique identifiers, or web links for publicly available datasets
- A list of figures that have associated raw data
- A description of any restrictions on data availability

Data that support the findings of this study are provided in the source data file of this paper and are available from the corresponding author upon reasonable request

Field-specific reporting

Life sciences study design

All studies must disclose on these points even when the disclosure is negative.

Sample size	Sample size was determined as the minimal number allowing non-parametric statistical analysis while complying with the 3Rs rule on reducing, replacing and refining the use of animals for scientific purpose.
Data exclusions	No data has been excluded from analysis.
Replication	Replicates were performed for all measurments within each assay (Duplicates for PCR, ELISA, ELISPOT and luminex).
Randomization	Hu-mice were distributed in experimental groups so that human cell reconstruction rates were homogenous between groups.<br>Convalescents NHPs were randomly assigned in two experimental groups. Additional six age matched (43.7 months +/-6.76) cynomolgus macaques from same origin were included in the study as controls naïve from any exposure to SARS-CoV-2.<br>For experiments that do not include animals, this is not relevant as they only include animal samples that were all tested at the same time.
Blinding	For security reason, animal ID and experimental group are indicated on the housing cage, thus Animals care, clinical examination and sampling was not blinded because constrains associated to BSL3 containement.<br>Cynomolgus macaque viral loads, CT scoring, IFNg ELISPOT, ICS, quantification of SARS-CoV-2 antibodies and quantification of antibody-induced inhibition of ACE-2 bindingwere determined blinded at CEA.

Reporting for specific materials, systems and methods

We require information from authors about some types of materials, experimental systems and methods used in many studies. Here, indicate whether each material, system or method listed is relevant to your study. If you are not sure if a list item applies to your research, read the appropriate section before selecting a response.

Materials & experimental systems

n/a	Involved in the study	
☐	☒	Antibodies
☐	☒	Eukaryotic cell lines
☒	☐	Palaeontology and archaeology
☐	☒	Animals and other organisms
☒	☐	Human research participants
☒	☐	Clinical data
☒	☐	Dual use research of concern

Methods

n/a	Involved in the study	
☒	☐	ChIP-seq
☐	☒	Flow cytometry
☒	☐	MRI-based neuroimaging

Antibodies

Antibodies used

anti-CD11b-V450 (ICRF44; BD; #560480; lot N°7062807 - dil. 1:20)
anti-CD11c-APC (3.9; BioLegend; #301614; lot N°B276681 - dil. 1:20)
anti-CD11c-BV605 (3.9; BioLegend; #301636; lot N°B285853 - dil. 1:20)
anti-CD137-APC (4B4; BD; #550890; lot N°9287986 - dil. 1:20)
anti-CD14-A700 (M5E2; BioLegend; #301822; lot N°B315064 - dil. 1:25)
anti-CD154-FITC (TRAP1; BD; #555699; lot N°8032873 - dil. 1:20)
anti-CD163-APC (GHI/61; BioLegend; #333610; lot N°B229767 - dil. 1:20)
anti-CD20-BV711 (2H7; BD; #563126; lot N°9197661 - dil. 1:20)
anti-CD3-APC-Cy7 (SP34-2; BD; #557757; lot N°9252411 - dil. 1:200)
anti-CD3-V500 (SP34-2; BD; #560770; lot N°0086782 - dil. 1:20)
anti-CD45-PerCP (D058-1283; BD; #558411; lot N°9171712 - dil. 1:40)
anti-CD4-BV510 (L200; BD; #563094; lot N°9182919 - dil. 1:33,3)
anti-CD4-FITC (L200; BD; #550628; lot N°9280194 - dil. 1:5)
anti-CD69-PE-Cy7 (FN50; BD; #557745; lot N°9136867 - dil. 1:10)
anti-CD80-BV786 (L307.4; BD; #564159; lot N°0100209 - dil. 1:20)
anti-CD86-BV605 (2331; BD; #562999; lot N°9301835 - dil. 1:20)
anti-CD8-BV650 (BW135/80; Miltenyi Biotec; #563821; lot N°8208777 - dil. 1:50)
anti-CD8-PE-Vio770 (BW135/80; Miltenyi Biotec; #130-113-159; lot N°5201007127 - dil. 1:50)
anti-HLA-DR-APC-H7 (L243; BD; #641411; lot N°0170972 - dil. 1:40)
anti-IFN-γ-V450 (B27; BD; #560371; lot N°9080787 - dil. 1:33,3)
anti-IL-13-BV711 (JES10-5A2; BD; #564288; lot N°9346015 - dil. 1:20)
anti-IL-17a-Alexa700 (N49-653; BD; #560613; lot N°9199405 - dil. 1:20)
anti-IL-2-PerCP5.5 (MQ1-17H12; BD; #560708; lot N°9294866 - dil. 1:10)
anti-TNF-α-BV605 (Mab11; BioLegend; #502936; lot N°B282176 - dil. 1:30,3)
anti-hCD3-A700 (UCHT1; sony; #2102120; lot N°ND - dil. 1:50)

anti-h-CD4-BV605 (RPA-T4; sony; #2102780; lot N°ND - dil. 1:10)
anti-hCD8-APC-Cy7 (SK1; sony; #2323570; lot N°ND - dil. 1:10)
anti-hIFNγ-PerCPCy5.5 (B27; sony; #3132640; lot N°ND - dil. 1:10)
anti-hIL-2-BV421 (MQ1-17H12; sony; #3101640; lot N°ND - dil. 1:16)
anti-hTNFα-PC7 (Mab11; sony; #3114650; lot N°ND - dil. 1:10)
anti-h-CD45-PeCy7 (HI30; sony; #2120080; lot N°ND - dil. 1:50)
anti-mouse-CD45-BV711 (30F11; sony; #1115735; lot N°ND - dil. 1:50)
anti-hCXCR4-Pe-Dazzle (12G5; eBiosciences; #12-9999-42; lot N°ND - dil. 1:50)
anti-hCCR10-PE (; R&D System; #314305; lot N°ND - dil. 1:50)
anti-CD3-BV510 (UCHT1; sony; #2102240; lot N°ND - dil. 1:50)
anti-CD4-FITC (OKT4; sony; #2187040; lot N°ND - dil. 1:50)
anti-CD8-PerCpCy5.5 (SK1; Biolegend; #2323550; lot N°ND - dil. 1:50)
anti-hCD19-BV421 (HIB19; Sony; #2111170; lot N°ND - dil. 1:16)
anti-hCD20-APC (2H7; sony; #2111550; lot N°ND - dil. 1:50)
anti-hIgG-BV786 (G18-145; BD; #564230; lot N°ND - dil. 1:16)
anti-hCD38-APC-Cy7 (HIT2; Sony; #2117670; lot N°ND - dil. 1:16)
anti-CD40.RBD (12E12, in house)

Validation

See the corresponding manufacturer datasheets on webpages for reference and validation. Crossreactivity of antibodies used in NHP experiments were confirmed using the "NHP reagents" online database : https://www.nhpreagents.org/ReactivityDatabase or on manufacturer datasheets in "reactivity " section.

Eukaryotic cell lines

Policy information about cell lines

Cell line source(s)

U2OS-ACE2 GFP1–10 or GFP 11 (in house, EMBO J (2020)39:e106267 https://doi.org/10.15252/embj.2020106267); U2OS cells (ATCC HTB-96) were obtained from the ATCC

Authentication

Cells lines were not authenticated

Mycoplasma contamination

CoAll cells are negative for mycoplasma contamination. Tests were performed on a monthly basis.

Commonly misidentified lines
(See ICLAC register)

No commonly misidentified cell lines were used in this study.

Animals and other organisms

Policy information about studies involving animals; ARRIVE guidelines recommended for reporting animal research

Laboratory animals

The study have included 21 cynomolgus macaques (Macaca fascicularis), aged 37-58 months (8 females and 13 males) and 29 female NSG (NOD.Cg-Prkdcscid Il2rgtm1Wjl/SzJ) humanized mice (hu-mice), aged 20-weeks. The hu-mice were housed in micro-isolators under pathogen-free conditions with human care, at a temperature of 20-24°C with 50% +/- 15% humidity and a 12-hour light/12-hour dark cycle.

Wild animals

No wild animals were used in this study

Field-collected samples

No field-collected samples were used in this study.

Ethics oversight

The mouse experimental study was approved by the institutional ethical committee “Comité d’Ethique Anses/ENVA/UPEC (CEEA-016)” under statement number 20-043 #25329. The study was authorized by the “Research, Innovation and Education Ministry” under registration number 25329-2020051119073072 v4.

Cynomolgus macaques (Macaca fascicularis), aged 37-58 months (8 females and 13 males) and originating from Mauritian AAALAC certified breeding centers were used in this study. All animals were housed in IDMIT facilities (CEA, Fontenay-aux-roses), under BSL-3 containment (Animal facility authorization #D92-032-02, Préfecture des Hauts de Seine, France) and in compliance with European Directive 2010/63/EU, the French regulations and the Standards for Human Care and Use of Laboratory Animals, of the Office for Laboratory Animal Welfare (OLAW, assurance number #A5826-01, US). The protocols were approved by the institutional ethical committee “Comité d’Ethique en Expérimentation Animale du Commissariat à l’Energie Atomique et aux Energies Alternatives” (CEtEA #44) under statement number A20-011. The study was authorized by the “Research, Innovation and Education Ministry” under registration number APAFIS#24434-2020030216532863v1.

Note that full information on the approval of the study protocol must also be provided in the manuscript.

Flow Cytometry

Plots

Confirm that:

- [x] The axis labels state the marker and fluorochrome used (e.g. CD4-FITC).
- [x] The axis scales are clearly visible. Include numbers along axes only for bottom left plot of group (a 'group' is an analysis of identical markers).
- [x] All plots are contour plots with outliers or pseudocolor plots.
- [x] A numerical value for number of cells or percentage (with statistics) is provided.

Methodology

Sample preparation	PBMC from 3 naïve macaques were isolated and stained for 15 min with antibody cocktail. Next, cells were washed twice with PBS before acquisition.<br>A part of these PMBCs were also incubated 18 hours with culture medium (RPMI 1640 media with L-Glutamax supplemented with Penicillin / Streptomycin and 10% of fetal calf serum (FBS)) and stimulated with αCD40.RBD (10 µg/mL) or LPS (100 ng/mL, Invivogen). Next, cells were washed in PBS and incubated 15min with antibody cocktail. Next, cells were washed twice with PBS before acquisition.<br><br>For Antigen specific T cell assays in NHP, one million of PBMC were cultured in complete medium (RPMI1640 Glutamax+, Gibco; supplemented with 10 % FBS), supplemented with co-stimulatory antibodies (FastImmune CD28/CD49d, Becton Dickinson). Then cells were stimulated with S or N sequence overlapping peptide pools at a final concentration of 2 µg/mL. Brefeldin A was added to each well at a final concentration of 10µg/mL and the plate was incubated at 37°C, 5% CO2 during 18 h. Next, cells were washed, stained with a viability dye (LIVE/DEAD fixable Blue dead cell stain kit, ThermoFisher), and then fixed and permeabilized with the BD Cytofix/Cytoperm reagent. Permeabilized cell samples will be stored at -80 °C before the staining procedure. Antibody staining was performed in a single step following permeabilization. After 30 min of incubation at 4°C, in the dark, cells were washed in BD Perm/Wash buffer then acquired.<br><br>Cryopreserved hu-mice spleen cells from 6 weeks after the priming immunization (one week after final immunization) were thawed and counted. Cells were rested overnight in RPMI 1640 media with L-Glutamax supplemented with Penicillin / Streptomycin and 10% of human serum. Subsequently, cells from HLA-A*0201 and HLA-A*0301 donors were pooled together for the mock group and group 2 plus 3 vaccinated hu-mice, then cultured at 0.6x106 cells per condition with 1µg/mL of 15-mers peptides JPT Peptide Technologies (Berlin, Germany). As a negative control no stimulant was added, and as a positive control 1 µL of DynabeadsTM CD3/CD28 (ThermoFischer Scientific) were used. IL-2 (100 IU/mL, R&D System) was added on day 2, half of the volume of each culture well was refreshed with fresh media containing IL-2 (10 U/mL) at day 5 and with fresh media without IL-2 at day 7. On day 8, cells were re-stimulated: no stimulant was added in the negative control, 100 ng/mL Staphylococcal enterotoxin B (LL-122, Cliniscience) was added in the positive control and 15-mers peptides in the condition of interest. BD GolgiPlug (Becton Dickinson France) was added in all conditions and the culture was continued for additional 18 hours. Next, spleen cells were washed using FACS buffer (PBS, supplemented with 1% FBS) and incubated with antibody cocktail.<br><br>Hu-mice PBMC from 3 weeks after the priming immunization and hu-mice PBMC and spleen cells from 6 weeks (one week after the last recall injection) were incubated first with the biotinylated SARS-CoV-2 S protein for 30 min at 4°C. After a washing step, cells were stained for 30 min at 4°C with antibody coktail. Staining on spleen cells also included a viability marker (LiveDead aqua or yellow stain ThermoFisher Scientific). Cells were washed twice with FACS buffer (PBS 1% FCS) and acquired.
Instrument	ZE5 flow cytometer (Biorad); LSRII flow cytometer (BD Biosciences)
Software	FlowJo v.10 and Flowjo V.10.7.1 software
Cell population abundance	No cell-sorting was performed in this study.
Gating strategy	First gating on "time" VS SSC-H was performed, then singlet were selected on SSC-W vs SSC-A gating. Alive cells were selected on SSC-A vs viability marker. Then lineage markers were used to analyze cell subset of interest.

- [x] Tick this box to confirm that a figure exemplifying the gating strategy is provided in the Supplementary Information.
